# Muscular Pre-activation Can Boost the Maximal Explosive Eccentric Adaptive Force

**DOI:** 10.3389/fphys.2019.00910

**Published:** 2019-07-23

**Authors:** Laura V. Schaefer, Frank N. Bittmann

**Affiliations:** Regulatory Physiology and Prevention, Department of Sports and Health Sciences, University of Potsdam, Potsdam, Germany

**Keywords:** Adaptive Force, neuromuscular pre-activation, power improvement, muscular activity, adaptation to external force impact

## Abstract

The improvement of power is an objective in training of athletes. In order to detect effective methods of exercise, basic research is required regarding the mechanisms of muscular activity. The purpose of this study is to investigate whether or not a muscular pre-activation prior to an external impulse-like force impact has an effect on the maximal explosive eccentric Adaptive Force (xpAFecc_max_). This power capability combines different probable power enhancing mechanisms. To measure the xpAFecc_max_ an innovative pneumatic device was used. During measuring, the subject tries to hold an isometric position as long as possible. In the moment in which the subjects’ maximal isometric holding strength is exceeded, it merges into eccentric muscle action. This process is very close to motions in sports, where an adaptation of the neuromuscular system is required, e.g., force impacts caused by uneven surfaces during skiing. For investigating the effect of pre-activation on the xpAFecc_max_ of the quadriceps femoris muscle, *n* = 20 subjects had to pass three different pre-activation levels in a randomized order (level 1: 0.4 bar, level 2: 0.8 bar, level 3: 1.2 bar). After adjusting the standardized pre-pressure by pushing against the interface, an impulse-like load impacted on the distal tibia of the subject. During this, the xpAFecc_max_ was detected. The maximal voluntary isometric contraction (MVIC) was also measured. The torque values of the xpAFecc_max_ were compared with regard to the pre-activation levels. The results show a significant positive relation between the pre-activation of the quadriceps femoris muscle and the xpAFecc_max_ (male: *p* = 0.000, η^2^= 0.683; female: *p* = 0.000, η^2^= 0.907). The average percentage increase of torque amounted +28.15 ± 25.4% between MVIC and xpAFecc_max_ with pre-pressure level 1, +12.09 ± 7.9% for the xpAFecc_max_ comparing pre-pressure levels 1 vs. 2 and +2.98 ± 4.2% comparing levels 2 and 3. A higher but not maximal muscular activation prior to a fast impacting eccentric load seems to produce an immediate increase of force outcome. Different possible physiological explanatory approaches and the use as a potential training method are discussed.

## Introduction

Classic considerations of force abilities do not include composed forms of muscle action, which are much more common in exercise performance and real life – irrespective of whether submaximal or maximal intensities and low or high velocities are performed. The composed forms arise from the basic muscle functions (concentric, eccentric, isometric), by combining at least two of them. In the field of high-speed performance, the SSC plays an important role, which consists of an eccentric muscle action followed by a concentric contraction (Komi, 1985, 2003; Schnabel et al., 2011).

**FIGURE 1:**
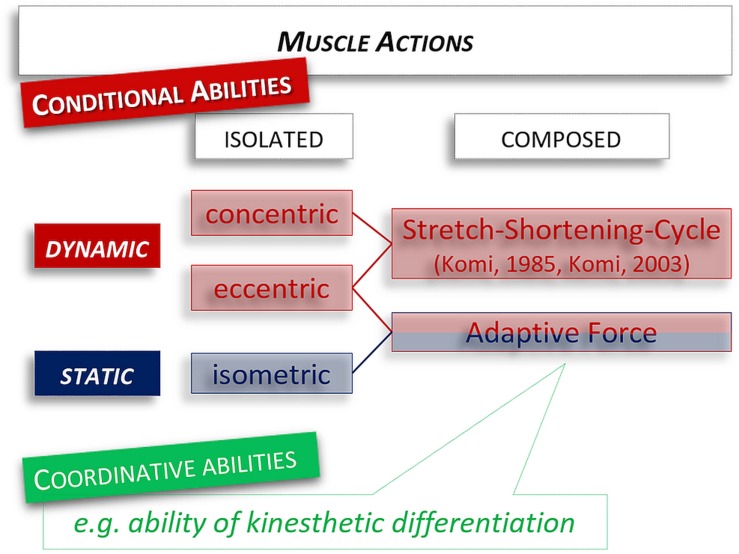
Classification of muscle actions. New is the addition of the composed muscle action with isometric-eccentric sequences – which corresponds to the explosive Adaptive Force used in this study. The specialty is that the possibility for the neuromuscular system to react and adapt to the external impacting force is considered.

Another possibility is to link an isometric with an eccentric muscle action. Thereby the isometric muscle activity is immediately followed by an eccentric action. We suggested to label this composed form of sensorimotor function the AF (Figure 1; Hoff et al., 2011, 2015; Schaefer et al., 2017). The feature of AF is the adaptation to varying external forces impacting on the body. Practical examples are walking down the stairs, receiving heavy objects into the hands, or – for the explosive AF – the absorption of uneven surfaces during skiing.

The aim during AF measurements is to hold the isometric position as long as possible, while an external increasing force impacts. AF can be performed with a slow or an explosive force increase. In the present study the focus lies on the explosive form (xpAF).

The xpAF has to be distinguished from the SSC, mainly because of the different sequences of basic muscle actions. During the SSC the task is to improve the power by switching from eccentric into concentric, nearly without an isometric phase. Whereas during AF the task is to adapt optimally to an external force while maintaining an isometric position as long as possible. If the external load exceeds the subjects’ maximal isometric holding force, it merges into eccentric muscle action. The adaptation process during AF is another distinct difference compared to the SSC.

The improvement of strength and power is an objective in training of athletes. Science and trainers are searching for training methods that still can improve or optimize the performance. Keywords such as eccentric training (e.g., Vogt and Hoppeler, 2014) or post-activated potentiation (e.g., Wilson et al., 2013) are just some to list. In order to detect effective methods of exercise, basic research is required regarding the mechanisms of muscular activity.

To mobilize additional strength reserves and, thereby, to improve the ability of voluntary muscle activation, it could make sense to combine the following regularities and principles of muscle physiology and biomechanics: (1) The principle of starting power (Hochmuth, 1974), (2) the achievement of highest forces through maximal isometric preload (Bührle et al., 1983; Linnamo, 2002; Linnamo et al., 2006), (3) the possibility to achieve supramaximal forces during eccentric action compared to concentric and isometric ones (e.g., Singh and Karpovich, 1966; Crenshaw et al., 1995; Kellis and Baltzopoulos, 1998; Reeves and Narici, 2003; Wirth and Schmidtbleicher, 2004; Shepstone et al., 2005; Meinel and Schnabel, 2007; Coadore et al., 2014), and (4) the proportionality between force and eccentric velocity (Schmidtbleicher et al., 1981; Röthing and Größing, 1990; Shepstone et al., 2005; Carney et al., 2012).

Bührle et al. (1983) explored the influence of maximal preload on the MVC as well as the eccentric force outcome using overload. Regarding this, Komi et al. (2000) did not find an improvement of eccentric muscle action performed with a maximal pre-activation measured by an isokinetic system compared to the maximal voluntary isometric contraction (MVIC). It has to be noted, that this result was found using the same angle at 110° during eccentric and isometric action. In a more detailed report of Linnamo (2002), it is described that the force outcome was highest at the eccentric motion with a maximal isometric pre-activation. But this was only the case without considering the angle. Thus, if the interest is on the maximum possible force outcome a muscle is able to produce, the consideration has to take all angular degrees into account.

In fact, it is well known that eccentric muscle action can generate higher forces than the isometric and concentric one (e.g., Singh and Karpovich, 1966; Crenshaw et al., 1995; Kellis and Baltzopoulos, 1998; Reeves and Narici, 2003; Wirth and Schmidtbleicher, 2004; Shepstone et al., 2005; Meinel and Schnabel, 2007; Coadore et al., 2014).

Another relevant factor concerning the force outcome seems to be the velocity of performed motion. Here again different findings were made. During concentric isokinetic measurements, the force of the elbow flexors in the middle angle position of 110° decreases significantly with higher velocity. During eccentric motion, no velocity dependence was identified (Komi et al., 2000; Linnamo, 2002). It has to be noted again, that the MVIC was compared at an angle of 110°. Immediately after beginning the eccentric motion at an elbow angle of 80°, the force exceeds the maximal isometric pre-activation. Furthermore, the higher the velocity of the eccentric motion, the more the force increased – but only with maximal pre-activation (Linnamo et al., 2006). This supports other investigations, in which high velocity eccentric motion induces even higher force outcomes compared to slow eccentric actions (e.g., Röthing and Größing, 1990; Shepstone et al., 2005; Carney et al., 2012). Some investigations show, in contrary, no velocity dependence (Westing et al., 1988; Chapman et al., 2005, 2006). In summary, there are inconsistent findings concerning the velocity-torque relationship in eccentric muscle action.

The purpose of this study was to investigate whether or not the extent of muscular pre-activation prior to a high-velocity eccentric overload has an influence on the xpAFecc_max_ of the quadriceps femoris muscle. It is hypothesized, that a higher pre-activation of the quadriceps femoris muscle is associated with a higher xpAFecc_max_. Thereby, two aspects are new. Firstly, the preload is nowhere near the MVIC, it is considerably lower. Secondly, the measurement of AF does not use a desmodromic system and combines two forms of the basic muscle actions: the performed isometric and subsequent eccentric muscle action depends on the individual ability to resist the external load. Real life motion normally does not proceed with forced velocities, as one can find it during eccentric measurements by means of an isokinetic system. Usually, the neuromuscular system is free to act against external forces in order to influence the movement speed. Since the measuring device used in the present study does not force the subject into particular velocities, we suppose that it is closer to real life motions.

## Materials and Methods

The study has an experimental design and took place in a laboratory of the University of Potsdam (dev. Regulatory Physiology and Prevention, Germany).

### Participants

The sample includes *n* = 20 healthy subjects: 19 sport students (male = 9, female = 10) of the University of Potsdam and one extern. The female subjects were aged averaged 22.2 ± 0.41 years (range: 22–23), the male subjects 25.9 ± 4.15 years (range: 22–37). 18 participants were not top-athletes; one female was an active competition athlete, one male was a former top-athlete. The subjects were practicing either endurance sports such as jogging, or power sports such as volleyball or basketball and others. Inclusion criteria were at least a 1-year freedom of complaints of the lower back and extremity. In the cases of two male subjects only the left leg could be included in the analysis because of knee problems during the trials on the right side.

Prior to the measurements the personal data, the sporting habits and the state of health of the participants were assessed through a questionnaire. Full verbal advice about possible risks and discomfort was given to the subjects.

### Strength Parameter: Adaptive Force

The concept of AF (Hoff et al., 2015; Schaefer et al., 2017) includes the ability of the neuromuscular system to adapt to an external increasing force: “AF means the specific behavior of adaptation of the nerve-muscle-system to external forces.” In submaximal intensities static conditions should be maintained (isometric: AFiso) and in supramaximal ranges the yield should be as slow as possible (eccentric: AFecc). Within this procedure, not only the force of the subject is required, but also the sensorimotor control of the neuromuscular system. The AF demands specific abilities of adaptation of the neuromuscular system in order to react adequately to the increasing external force, which is applied to the individual.

### Measurement System and Procedure to Record the Adaptive Force

In a project supported by the German Federal Ministry of Economy (ZIM KF2262301FO9) a pneumatic system was constructed and evaluated to measure inter alia the quadriceps femoris muscle. The benefit of the use of pneumatics is that the subject is able to hold an isometric position in submaximal intensities. Afterward, when the individual maximal isometric AF is overcome, the muscle action passes into eccentric.

#### Measuring System

**FIGURE 2:**
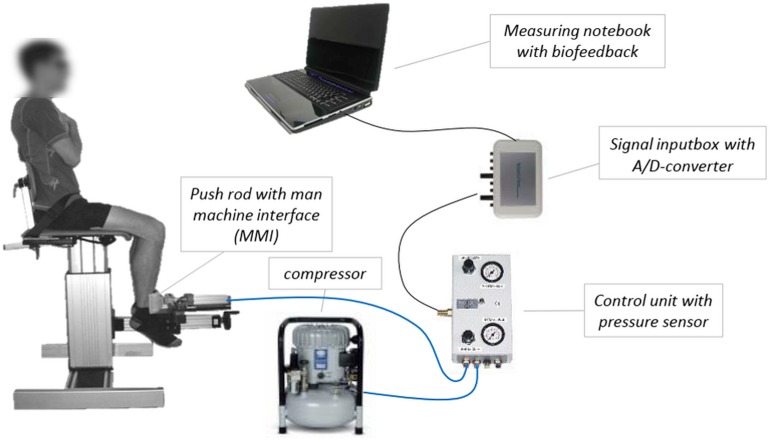
Schematic diagram of the pneumatic measuring system “*SeBit knee*.” The push rod of the cylinder (∅ 12.57 cm^2^) is equipped with a man machine interface. The cylinder is connected with a compressor (Jun-Air, Agre Kompressor Hobby Star 200, max. 8 bar), which can be controlled by a control unit. The input-box including an A/D-converter (14-bit) transmits the signal of the pressure sensor to the measuring notebook containing the software NI DIAdem 10.2. The value of pressure serves as biofeedback to adjust the pre-pressure during the measuring of explosive Adaptive Force (xpAF) (Hoff et al., 2015; Schaefer et al., 2017).

**FIGURE 3:**
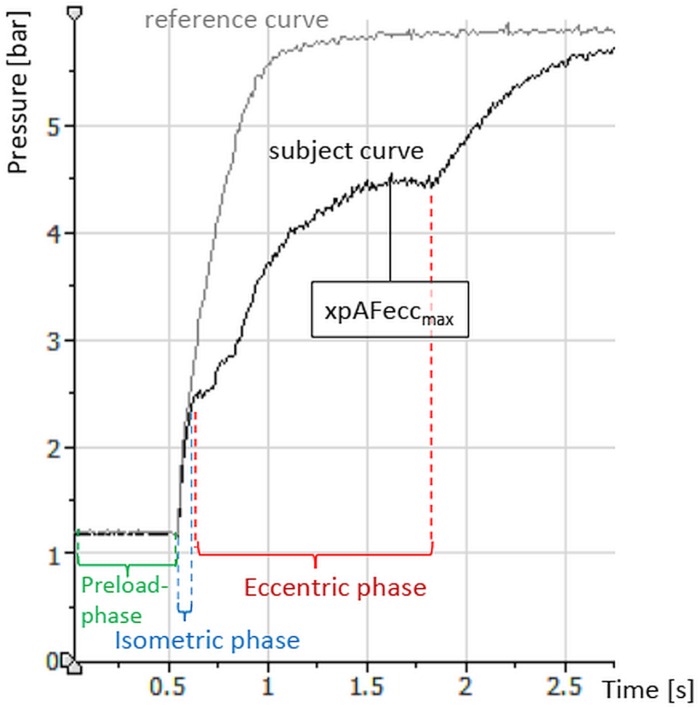
Exemplary curves (raw data) of the explosive Adaptive Force comparing one trial against a stable resistance (reference, gray) and one trial of a subject with the same adjustments in pressure increase and pre-load. Displayed are, furthermore, the relevant phases of the measurement process.

Figure 2 illustrates the pneumatic system *SeBit knee*, which is used to measure the AF of the quadriceps femoris muscle. The control unit allows the adjustment of different pre-pressures as well as various velocities of pressure rise via choke. Thereby, different slew rates can be configured, so that the basic form of AF – whereby the force increases slowly – as well as the xpAF – characterized by an impulse-like pressure increase – can be measured by the same system. In this study, the xpAF is the parameter of interest. For measuring the xpAF, the system pressure is chosen at 6 bar [force: 754 N; with averaged lever length of 0.3 m (±0.2) a torque of 226 Nm]. For these measurements, the choke is opened completely, so that the pressure of 6 bar is achieved within approximately 1 s during a measurement against a complete stable resistance (see reference curve in Figure 3).

A pressure sensor in the control unit records the pressure. This analog signal is forwarded via an A/D-converter to the measuring notebook with software NI DIAdem 10.2 (National Instruments). The software stores the data and opens the possibility to edit them.

#### Measuring Process

The xpAF is characterized by an impulse-like increase of pressure. Furthermore, during measurement three phases can be distinguished (Figure 3): (1) The *preload phase*: a short submaximal isometric phase of around 0.5–1 s, whereby the amount of pre-activation can be selected. (2) The *isometric phase*: the amount of force increases while the subject must maintain the isometric position as long as possible. (3) The *eccentric phase*: the force increase is so high that the maximal isometric AF of the subject is overcome. The muscle merges into eccentric work, it yields in its length. Thereby, the maximal value corresponds to the xpAFecc_max_, which is the parameter of interest here.

The effect of different muscular pre-activations on the xpAFecc_max_ was examined for the quadriceps femoris muscle with the system *SeBit knee* mentioned above (Figure 2). To investigate the hypothesis that a higher pre-activation has an effect on the force outcome of the xpAFecc_max_, three levels of pre-pressure of the pneumatic system were used: Level 1: 0.4 bar (=̂50 N); Level 2: 0.8 bar (=̂101 N); Level 3: 1.2 bar (=̂151 N). The subject adjusted the level of pre-pressure by pressing against the MMI-interface.

Five trials per level of pre-pressure were done on each side. Both sides were measured consecutively. The trials were arranged in such a way that randomly the half of the subjects (*n* = 10) passed the levels of pre-pressure from bottom to top, while the other half (*n* = 10) passed them from top to bottom. In this way any effect of the order of pre-pressure on the force outcome, e.g., fatigue, should be eliminated. The resting period between the trials was 30 s. Since the interval of muscular activation lasts approximately 3 s, a break of 30 s should be enough for recovery.

Prior to the measurements the sitting position of each subject was adjusted. The system *SeBit* allows an adjustment of the position in all three dimensions. The subject was positioned with a 90° hip and knee angle in the starting position. During measurement, the arms of the subject should be crossed on the chest. To avoid a force recruitment through the low backrest, the subject had to sit up. The MMI was applied from ventral with its lower edge at the distal tibia 2 cm above the medial malleolus.

A trial started with the adjustment of the given level of pre-pressure (0.4, 0.8, or 1.2 bar, respectively) performed by the subject using a biofeedback via notebook display. Thereby, the subject pushed the push rod with its distal tibia into the cylinder until the given pre-pressure level is reached (knee angle then 90°). This isometric position should be maintained for as long as possible during the following measuring process. Approximately 1 s after the adjustment of pre-pressure the pressure increase was executed. Since the pressure increases impulse-like in supramaximal areas, the subject is only able to hold the isometric position for a split second; afterward it merges into eccentric muscle action.

After a total of 15 measurements of xpAF, three trials of MVIC of each leg were performed by pushing maximally within 3 s against the interface of the pneumatic cylinder, which at first yields slightly and then develops a stable resistance. In this isometric position the knee angle amounted to 90°. The resting period between the MVIC trials was 2 min. The difference with respect to the xpAF trials is that the subject pushes maximally against the stable resistance (common method to record MVIC), whereby while performing the xpAF trials, it has to react and adapt to the impacting force by holding isometrically. The decisive difference lies in the distinction of a holding and pushing isometric muscle action, which was reported earlier (Schaefer and Bittmann, 2017).

### Data Processing

**FIGURE 4:**
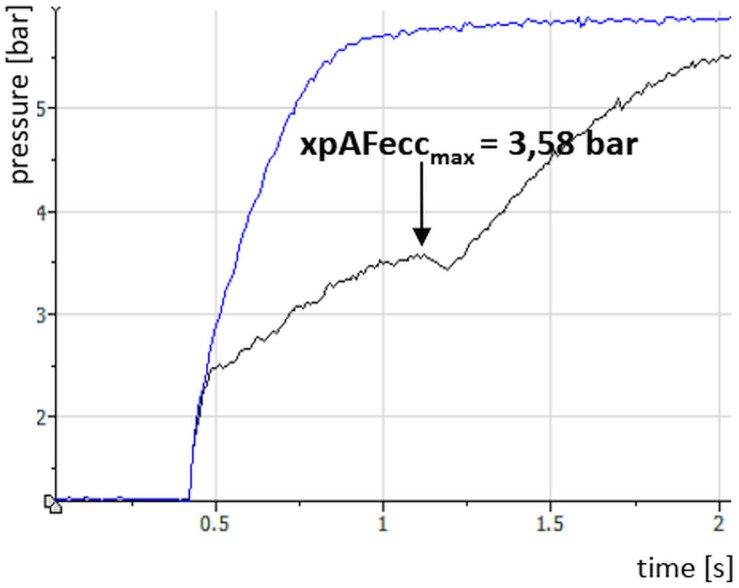
Displayed is a calibration curve (blue) and a measuring curve (black) with a pre-pressure of 1.2 bar and an impulse-like pressure increase. The maximal value represents the maximal explosive eccentric Adaptive Force (xpAF_max_).

The main data processing was performed with the Software NI DIAdem 10.2. The aim was to determine the highest value of the pressure curve during the eccentric phase (xpAFecc_max_; Figure 4), regardless the angle of knee joint. In order to ensure the values and, thus, to assure reliability regarding the evaluation of the xpAFecc_max_ two raters independently analyzed the raw curves. Trials with an adjustment of the pre-pressure level longer than 5 s have been excluded (in total 12 of 300 trials). In 20 of 300 trials, the xpAFecc_max_ value could not be identified, since no maximum was present in the pressure curve. The arithmetic means and SDs of xpAFecc_max_ of the five trials per pre-pressure level as well as of the three MVIC trials were calculated. For descriptive statistics, the torque was calculated using the maximal pressure values [bar], the individual lever length (distance between the middle of the condyles of femur and the middle of MMI) and the diameter of the cylinder: 12.57 cm^2^). The percentage difference (%-diff) between the MVIC and the xpAFecc_max_ values (regarding the pre-pressure levels 0.4, 0.8, and 1.2 bar) was calculated, whereby the MVIC was set at 100%. The following formula was used: $\%-\mathrm{diff}=\frac{\mathrm{xpAFecc}}{\mathrm{MVIC}}*100-100$. Hereby, xpAFecc stands for the arithmetic mean of the values of xpAFecc_max_ of five trials of the respective pressure level and MVIC represents the arithmetic mean of the three MVIC trials.

Using SPSS 25, the data were checked for normal distribution with the Shapiro–Wilk test. Subsequent, the torque values were differentiated by gender and compared between the groups of MVIC, xpAFecc_max_ 0.4, 0.8, and 1.2 using the ANOVA with repeated measures, since data were parametric. The assumption of sphericity, tested by Mauchly’s test, has been violated. The Greenhouse–Geisser correction was used. For *post hoc*-testing the Bonferroni test was used. The effect size was calculated using the partial eta squared. For further comparisons the percentage differences between the four groups (MVIC, pre-pressure levels 1–3; arithmetic mean of left and right) were analyzed concerning factors including gender, starting leg (left or right), order of pre-pressure (low-high or high-low) and type of sport (endurance or power). Here also the Shapiro–Wilk test was used to verify normal distribution. The Levene’s test was utilized to examine the homogeneity of variances. The variances of the percentage differences between each level of the xpAFecc are homogeneous, thus a MANOVA was done incl. a Bonferroni correction. Since the variances of the percentage differences between the MVIC and the xpAFecc were not homogeneous, the non-parametric Mann–Whitney *U* test was performed for group comparisons. The effect size was calculated using partial eta squared and Pearson’s *r*. Significance level was always set at α = 0.05.

## Results

### Curve Characteristics

**FIGURE 5:**
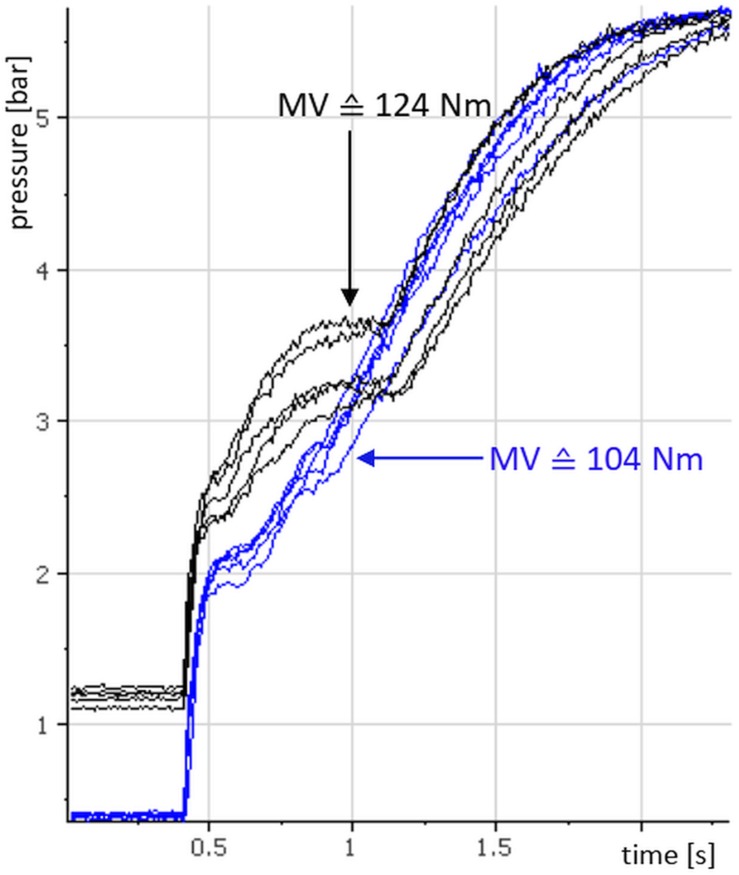
Pressure curves of the explosive Adaptive Force (xpAFecc) of one subjects’ left quadriceps femoris muscle: illustrated are each five repeated trials with a pre-pressure of 0.4 bar (blue) and with a pre-pressure of 1.2 bar, respectively. The arithmetic mean values of xpAFecc_max_ are 2.85 bar (103.96 Nm) for the pre-pressure of 0.4 bar and 3.42 bar (124.49 Nm) for the pre-pressure of 1.2 bar, respectively.

Figure 5 shows 10 raw curves of one subject’s xpAFecc of the left quadriceps femoris muscle: five at a pre-pressure of 0.4 bar (blue) and five at a pre-pressure of 1.2 bar (black). The pre-activation phase is cut here to an interval of 0.4 s.

The isometric phase is very short (about 0.05 s) due to the impulse-like pressure increase. The eccentric period lasts approximately 0.4 s during the trial with a pre-pressure of 0.4 bar, whereas in the trial with a pre-pressure of 1.2 bar the eccentric period lasts approximately 0.6 s. The following rise of the graph reflects the increase of system pressure after the cylinder piston is completely moved out. That means that the subject is no longer able to resist the increasing load. The arithmetic mean values of xpAFecc_max_ (Nm) in this example amount 104 Nm for the lower pre-activation with 0.4 bar and 124 Nm for the higher pre-pressure of 1.2 bar. Thus, in this example there is an increase of xpAFecc_max_ between pre-pressure 0.4 and 1.2 of approximately 20%.

### Comparison of Torque Values of MVIC and the Pre-activation Levels

Arithmetic mean (±SD) of the torque values of the MVIC and of xpAFecc_max_ values at each pre-pressure level of all subjects and in total are displayed in Table 1.

**TABLE 1:** Arithmetic mean and standard deviation of torque values (Nm) of the MVIC and the xpAFecc_max_ at pre-pressure levels 0.4, 0.8, and 1.2 of each subject.

			**MVIC(Nm)**	**xpAFecc_max_ (Nm)**		
				**0.4**	**0.8**	**1.2**
Male	1	Left	120.42 ± 7.42	197.05 ± 2.86	200.01 ± 3.75	204.86 ± 3.19
		Right	151.57 ± 10.08	184.85 ± 7.97	193.24 ± 7.73	203.63 ± 4.75
	2	Left	91.54 ± 1.20	130.61 ± 2.03	141.63 ± 2.88	147.90 ± 5.31
		Right	110.86 ± 0.37	141.47 ± 21.30	150.39 ± 3.40	148.25 ± 7.05
	3	Left	124.79 ± 3.12	143.05 ± 4.99	147.09 ± 3.19	149.58 ± 5.11
		Right	140.40 ± 2.36	154.77 ± 4.14	162.90 ± 3.18	169.23 ± 7.12
	4	Left	131.80 ± 5.66	180.40 ± 3.57	176.54 ± 4.48	165.87 ± 6.73
		Right	134.85 ± 2.08	158.74 ± 6.15	160.12 ± 4.02	169.20 ± 4.61
	5	Left	95.94 ± 2.78	97.96 ± 3.71	116.39 ± 5.76	117.52 ± 7.45
		Right	99.67 ± 4.23	115.67 ± 8.56	126.07 ± 4.51	127.26 ± 4.89
	6	Left	79.43 ± 4.01	121.86 ± 11.14	137.51 ± 2.40	138.19 ± 5.61
		Right	95.51 ± 1.98	132.25 ± 21.23	139.01 ± 5.90	142.93 ± 9.05
	7	Left	104.85 ± 5.71	103.96 ± 9.03	120.55 ± 3.78	124.49 ± 7.91
		Right	120.82 ± 4.30	86.50 ± 2.49	111.56 ± 2.59	129.11 ± 6.17
	8	Left	117.63 ± 2.69	136.74 ± 2.94	145.89 ± 5.64	150.37 ± 4.33
		Right	120.34 ± 9.35	143.87 ± 5.50	145.95 ± 6.94	149.45 ± 6.73
	9	Left	137.08 ± 6.04	128.55 ± 12.35	165.38 ± 4.91	179.32 ± 2.70
		Right	120.92 ± 5.87	144.00 ± 6.83	160.57 ± 4.19	168.68 ± 2.57
	10	Left	105.39 ± 6.19	124.11 ± 5.08	138.70 ± 16.86	142.34 ± 2.33
		Right	125.69 ± 6.13	118.41 ± 2.07	137.84 ± 3.52	129.33 ± 12.90
	**Total**		**116.48 ± 18.43**	**137.24 ± 28.30**	**148.87 ± 23.37**	**152.88 ± 24.35**
Female	11	Left	92.89 ± 7.48	102.44 ± 9.61	119.40 ± 3.11	128.09 ± 2.93
		Right	86.40 ± 9.14	96.90 ± 7.87	108.76 ± 7.46	121.11 ± 4.22
	12	Left	59.63 ± 4.48	97.85 ± 10.64	111.98 ± 4.24	113.67 ± 2.11
		Right	51.30 ± 3.00	97.93 ± 5.62	105.39 ± 8.66	106.90 ± 5.96
	13	Left	69.36 ± 7.12	94.90 ± 5.01	106.12 ± 3.83	112.80 ± 7.80
		Right	78.70 ± 6.21	104.80 ± 7.95	126.07 ± 3.35	123.03 ± 6.84
	14	Left	67.32 ± 6.51	91.34 ± 3.11	106.06 ± 2.97	114.49 ± 6.29
		Right	67.82 ± 2.53	89.29 ± 3.85	111.18 ± 5.26	116.09 ± 4.57
	15	Left	68.72 ± 2.23	108.81 ± 4.43	113.27 ± 2.66	110.68 ± 2.41
		Right	51.77 ± 2.34	95.29 ± 3.47	101.50 ± 4.75	103.21 ± 4.48
	16	Left	66.61 ± 5.47	107.67 ± 2.74	117.60 ± 3.21	116.55 ± 3.02
		Right	66.29 ± 3.14	111.99 ± 4.35	119.30 ± 1.49	118.00 ± 2.46
	17	Left	86.59 ± 2.21	88.15 ± 2.58	110.80 ± 3.32	118.27 ± 4.36
		Right	84.16 ± 0.24	95.99 ± 16.24	114.82 ± 2.33	118.88 ± 3.96
	18	Left	89.79 ± 1.68	118.52 ± 3.67	129.71 ± 5.15	131.54 ± 6.35
		Right	99.74 ± 1.58	111.21 ± 8.76	129.86 ± 5.23	140.98 ± 8.87
	19	Left	89.76 ± 2.52	115.71 ± 5.55	122.70 ± 4.65	124.74 ± 4.23
		Right	80.77 ± 8.19	98.66 ± 4.20	116.97 ± 4.93	119.25 ± 4.73
	20	Left	93.39 ± 4.95	105.87 ± 6.09	124.17 ± 5.74	127.94 ± 4.05
		Right	79.47 ± 4.56	112.22 ± 4.81	136.42 ± 3.61	138.54 ± 4.60
	**Total**		**76.53 ± 14.03**	**102.09 ± 8.79**	**116.60 ± 9.39**	**120.24 ± 9.67**

The group comparisons of the four parameters (MVIC, xpAFecc_max_ at 0.4, 0.8, and 1.2 bar) differentiated according to gender are significant in the ANOVA with repeated measures. (male: *F*(3,57) = 40.916, *p* = 0.000, η^2^= 0.683; female: *F*(3,57) = 184.92, *p* = 0.000, η^2^ = 0.907). The *post hoc* Bonferroni test delivers the highest *p*-value for the comparison of xpAFecc_max_ at pre-pressure levels 2 and 3 in the male as well as in the female group. The male group is no longer significant in this comparison with *p*_adjust_ = 0.76. The female groups is still significant with *p*_adjust_ = 0.008. All other comparisons are significant with *p* = 0.000.

### Percentage Difference Between MVIC and the Pre-activation Levels

**FIGURE 6:**
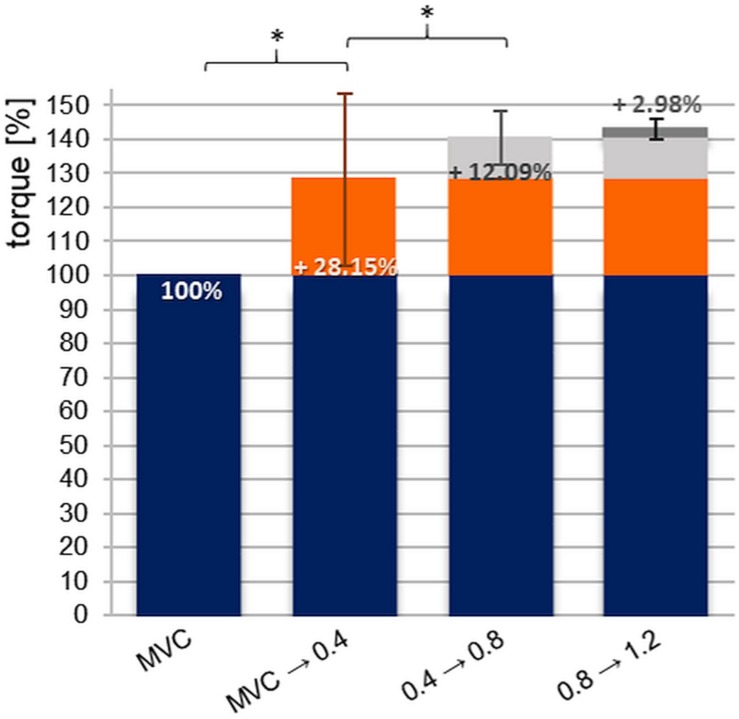
Displayed are the percentage differences (%-diff) between the averaged MVIC and the values of xpAFecc_max_ with respect to the pre-pressure levels 0.4, 0.8, and 1.2 bar of all *n* = 20 subjects. The MVIC level was set at 100%. The bar “MVIC → 0.4” indicates the %-diff of values of MVIC and xpAFecc_max_ at a pre-pressure of 0.4 bar (^*^p_adj*ust*_ = 0.026, *r* = 0.13); “0.4 → 0.8” stands for the %-diff of xpAFecc_max_ comparing pre-pressure levels 0.4 and 0.8 bar (^*^p_adj*ust*_ = 0.000, *r* = 0.19); “0.8 → 1.2” displays the %-diff of xpAFecc_max_ values comparing pre-pressure levels 0.8 and 1.2 bar (*p*_adj*ust*_ = 0.116). The error bars illustrate the standard deviation of the related %-diff.

Figure 6 shows the average percentage difference between MVIC and the three pre-pressure levels, whereby the MVIC was set as 100%. Between the values of MVIC and xpAFecc_max_ at pre-activation level 1 (0.4 bar) an average difference of +28.15 ± 25.4% occurs (range: −28.4 to +90.9%; male: 18.8 ± 21.5%, female: 37.5 ± 26.0%). The difference of torque between pre-activation level 1 and 2 provide an increase of +12.09 ± 7.9%; range: −2.1 to +29.0%; male: 9.73 ± 8.6%, female: 14.5 ± 6.6%). Between pre-pressure levels 1 and 3 (1.2 bar) there is a rise of averagely +15.58 ± 11.2%; range: −8.1 to +49.3%; male: 12.97 ± 12.56%, female: 18.2 ± 9.34%). The rise between level 2 (0.8 bar) and 3 (1.2 bar) amounts on average +2.98 ± 4.2% (range: −6.2 to +15.7%; male: 2.8 ± 4.7%, female: 3.2 ± 3.8%).

**FIGURE 7:**
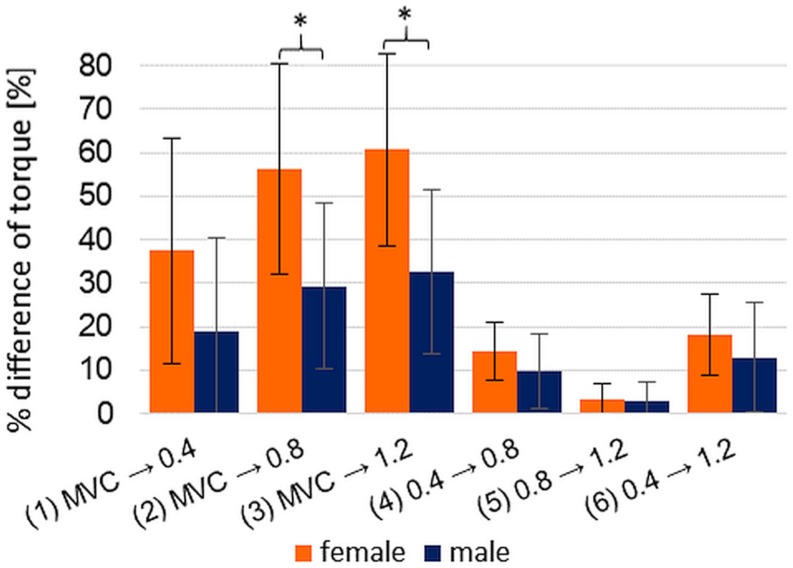
Displayed are the gender specific arithmetic means (±SD) of the percentage difference of each torque comparison: MVIC to xpAFecc_max_ per pre-pressure 0.4 bar (1), 0.8 bar (2), and 1.2 bar (3), respectively, and xpAFecc_max_ compared within the pre-pressure levels [0.4 → 0.8 bar (4), 0.8 → 1.2 bar (5), 0.4 → 1.2 bar (6)]; The comparisons between male and female (each *n* = 10) show partly significant differences: ^*^p = 0.005.

The percentage differences of torque between the pre-pressure levels of xpAFecc_max_ show no significance in the MANOVA concerning the factors type of sport, starting leg and order of pre-pressure levels (0.4 → 0.8: *F*(14,5) = 0.708; *p* = 0.721; η^2^_corrected_ = −0.275; 0.8 → 1.2: *F*(14,5) = 1.426; *p* = 0.368; η^2^_corrected_ = 0.239; 0.4 → 1.2: *F*(14,5) = 0.922; *p* = 0.589; η^2^_corrected_ = −0.061). Regarding the percentage differences of torque between MVIC and the pre-pressure levels 2 and 3 of xpAFecc_max_, the Mann–Whitney *U* test delivers a significant result concerning the gender (MVIC vs. xpAFecc_max_ with 0.8 bar: *U* = 14.00, *p* = 0.005; MVIC vs. xpAFecc_max_ with 1.2 bar: *U* = 14.00, *p* = 0.005), whereby the female show a significantly higher torque increase (diff MVIC – xpAFecc_max_ with 0.8 bar: female: 55.77 ± 18.74; male: 28.55 ± 16.04; diff MVIC – xpAFecc_max_ with 1.2 bar: female: 60.17 ± 20.20; male: 31.90 ± 15.72) (Figure 7). The other factors have no significant impact on the percentage difference between MVIC and the pre-pressure levels of xpAFecc_max_.

In summary, the results suggest that there is a positive relation between a pre-activation of the quadriceps femoris muscle and the xpAFecc_max_. A higher but not maximal muscular activation prior to an impulse-like impacting eccentric load seems to produce an acutely higher force outcome.

## Discussion

The aim of this study was to investigate whether or not a pre-activation of the quadriceps femoris muscle prior to an impulse-like impacting external load can increase the force outcome. The xpAFecc_max_ was examined, because it implements specific regularities and principles of muscle physiology, which are associated with a higher force outcome. Furthermore, the measuring enables the individuals a more or less self-controlled adaptation to the external force impact of the pneumatic device.

The results lead to the assumption of the following causality: A higher but not necessarily maximal pre-activation (approximately 16 to 47% of the MVIC) of the quadriceps femoris muscle enhances the output of the xpAFecc_max_. Before this can be accepted as a conclusion, possible methodological limitations should be considered.

### Methodological Limitations

The measured force values could be influenced by changes of the measuring position, varying air pressure of the pneumatic system or differing slew rates of the external force produced by the cylinder. By means of a strictly defined and documented procedure these factors were controlled. In addition, the recorded amounts and slew rates of the air pressure were checked afterward. Another point to take into account could be the influence of a possible fatigue appearing during the course of trials. This should be avoided by the randomized order of pre-pressure levels. Fatigue due to the pre-pressure load can also be ruled out because of the shortness of the pre-activation periods. Furthermore, one could discuss a probable habituation effect in the course of the repeated trials. This also appears to be unlikely because of the reverse sequences of levels. Eventually, there is the question of a possible measurement bias. This aspect can be largely excluded. The subjects were instructed about the pre-pressure levels, but they were not aware of the particular research question.

A weakness of the present study is the abovementioned non-individualized pre-pressure levels. They were generally chosen at the 0.4, 0.8, or 1.2 bar. The xpAFecc_max_ could probably be further enhanced by an individualized pre-activation, e.g., by using a specific percentage of the MVIC. The velocity of the force impact could possibly be optimized, too.

Regarding the results related to the MVIC, one has to take into account that the MVIC was measured after the trials of xpAFecc_max_. Probable fatiguing effects could falsify the amount of MVIC. Thus, the percentage increase between MVIC and xpAFecc_max_ might be lower than found in the present study. However, the enhancement of xpAFecc_max_ between the different pre-pressure levels is not affected by this.

The following content-related discussion deals with three main considerations: (1) related phenomena and influencing factors, (2) physiological explanatory approaches, and (3) probable application in exercise.

### Related Phenomena and Influencing Factors

#### Related Phenomenon: Post-activation Potentiation

A similar and probably related effect of strength and power enhancement is known as the PAP. Referring to this, the performance can be improved acutely but transient by a preceding voluntary contraction (e.g., Brown and Loeb, 1998; Hodgson et al., 2005; Tillin and Bishop, 2009; Lesinski et al., 2013; Wilson et al., 2013). PAP is triggered by a voluntary contraction with usually maximal or high intensities. This entails a higher peak force at subsequent contractions. The effect depends on different variables, such as training experience and resting period between conditioning activity and power output (e.g., Wilson et al., 2013). Furthermore, it works only for fast twitch muscle fibers. Investigations of PAP are usually performed concerning isometric or concentric muscle actions (Sale, 2002). Eccentric seems not to play a role regarding PAP. Therefore, no statement can be made concerning effects of PAP on eccentric muscle action. Some relevant differences between PAP and the boosting effect found in the present study must be considered: Firstly, using PAP as method of power and strength enhancement in performance, there has to be a resting period between activation and the desired raise in power. In the present study, an external explosive force impacts on the preloaded muscle and directly leads to an increase in power outcome. Secondly, the pre-activation in PAP must be higher for the enhancing effect than the here used pre-pressures. And thirdly, the power enhancements due to PAP are lower than in the presented investigation: the power change after activation varies in a range between −5.3% (decrease) up to +7% (Lesinski et al., 2013). Nevertheless, some assumed explanations for the physiological mechanisms of PAP can possibly be transferred to the boosting effect of pre-pressures on the xpAFecc_max_. Firstly, the phosphorylation of myosin regulatory light chains and, secondly, an increase in the recruitment of higher order motor units are discussed for PAP (Hodgson et al., 2005; Tillin and Bishop, 2009) (see next chapter).

#### Influencing Factor: Velocities of Force

The power enhancement after higher muscular pre-activation seems to depend on the velocity of the subsequent impacting force. In a pilot-study (*n* = 8) at our department, the “boosting” effect was not apparent during slow pressure rise. The “normal” maximal eccentric AF with a slow pressure rise was generally lower compared to the xpAFecc_max_ with an impulse-like pressure rise. Moreover, the amount of pre-pressure had no influence on the slow AFecc_max_. Due to the small sample size, this only can be seen as first hints. The rise of pressure during the xpAF is so impulsive that a contractile stretch reflex is possibly triggered, which originates from the muscle spindles (see below). This would likely not be present in the slow AFecc_max_.

Nevertheless, the results concerning velocity dependence are not exactly in accordance with the investigation of Linnamo et al. (2006). They only found a velocity-dependent force increase by executing a pre-activation level of 100% of the MVC, not at 50% of the MVC. Our findings suggest that a lower pre-activation level can also increase the force outcome during a high-velocity eccentric force impact. This disparity could probably lie in the different measuring systems: in performing the AF the neuromuscular system initially has the possibility to adapt to the external force in an isometric manner prior to the eccentric yielding. This adaptation process does not occur during isokinetic measuring due to the desmodromic procedure.

#### Influencing Factor: Level of Pre-activation

The results show that there is no need of a maximal muscular pre-activation to increase the force outcome. Investigations of other authors reveal higher or maximal pre-activation to generate a force increasing effect (Schmidtbleicher et al., 1981; Bührle et al., 1983; Linnamo, 2002). Since averagely 42.73 ± 25.48% of force rise already was achieved at a pre-pressure of 0.8 bar, obviously the enhancing effect already appears at a pre-activation amount of averagely 31.5 ± 8.1% of the MVIC. Though, a submaximal activated muscle prior to the supramaximal force impact suffices to produce a higher power outcome. The further rise of xpAFecc_max_ between the pre-pressure levels of 0.8 bar and 1.2 bar is substantially lower, although still statistically significant in female. The question is whether or not there still could be a considerable rise of xpAFecc_max_ with even higher pre-pressures. It must be assessed, if such intensities would be necessary concerning probable applications of the xpAF.

### Physiological Explanatory Approaches

Although our results suggest a relation between muscular pre-activation and force outcome, they do not reveal information related to the physiological mechanisms behind it. Therefore, only theoretical considerations will be done in the following.

#### Viscoelastic Properties and Passive Muscle Stiffness

The phenomenon of force increase due to muscular pre-activation could be influenced by muscle stiffness. Stiffness is defined as the passive mechanical properties of muscle, which can be expressed in the relationship between passive resistive torque and joint displacement (Herda et al., 2011). Various authors investigated muscle stiffness after eccentric exercise and proposed that stiffness increases after exercise due to the produced muscle damages (e.g., Howell et al., 1993; Whitehead et al., 2001; Green et al., 2012). In our setting, nothing comparable should have occurred, since no eccentric training was applied.

In a review of *in vitro* studies, Rassier (2012) reported that after stretch of an activated sarcomere, the sarcomeres’ residual force was enhanced. To explain this, he suggests the following mechanisms: (1) non-uniformity of half-sarcomeres which are influencing the stiffness of titin and the overlap between actin and myosin, and (2) a Ca^2+^-regulated increase in stiffness during activation and stretch. With this proposal, the passive stiffness of the myofibril gets higher with higher activation. This effect was not velocity dependent. Nevertheless, it could probably explain the force enhancement with higher pre-activation. The question remains as to whether or not these *in vitro* circumstances can be transferred to real life motions. Furthermore, the phenomenon found in the present study seems to be velocity dependent.

A reasonable explanation for this could result from an ordinary physical phenomenon: the higher the velocity of muscle activation the higher the resistance with constant viscosity. Thus, with higher velocity the resistance in the muscle fibers becomes more relevant and, therefore, the viscosity in the plasma of the muscle fibers could be a reason for the “boosting” effect.

#### Mechanisms of Motor Control

Another explanation for the force-enhancing effect of muscular pre-activation could lie in the interaction of different mechanisms of motor control.

Enoka (1996) supposes that eccentric muscle activation involves different and more complex neurological strategies of the organism than concentric action. One difference between concentric and eccentric is the suppression in muscular activity during eccentric. The specific neural pathways which are responsible for the differences between concentric and eccentric remain unidentified (Aagaard and Mayer, 2007; Duchateau and Enoka, 2008).

DeLuca et al. (1982) and other investigators (e.g., Linnamo et al., 2003) postulate that recruitment is the major mechanism to generate extra forces between 40 and 80% of the MVC. This leads to the assumption that at a pre-pressure of 1.2 bar (=47% of the MVIC) additional motor units would have been recruited. Since above 80% of the MVC the recruitment of additional muscle fibers is more and more exhausted, other mechanisms of motor control like synchronization or firing rates could play a major role. One can assume that synchronization and/or higher firing rates cause a greater increase of the supramaximal neuromuscular activity if they are based on a larger number of muscle fibers which are activated at increased pre-pressure.

#### Stretch-Induced Reflex and Higher Activation of Motor Units

Ishikawa and Komi (2007) reported that the stretch-induced reflex can play an important role in regulating the stiffness of muscle fibers. Therefore, it might be reasonable to suspect that the mechanism of stretch reflex is an influencing factor of the xpAF.

It is supposed that a higher pre-activation prior to the performance of the xpAFecc_max_ entails a higher force output, since a higher number of motor units are already activated. This is underpinned by the investigations of Mynark and Koceja (1997). They postulate that the H-reflex (peak-to-peak) increases during higher forces (10, 20, 30% of the MVIC). In the present study, the pre-activation of muscle amounts averagely 16% of the MVIC for a pre-pressure of 0.4 bar, 31% for 0.8 bar and 47% for a pre-pressure of 1.2 bar, respectively. Possibly, the stretch reflex hereby also increases with higher pre-activation. The influence of this neuronal factor could probably play an important role for the mechanisms of the xpAF. This would also be consistent to the fact that the force enhancing effect appeared only at high velocities. Schmidtbleicher et al. (1981) also proposed, that only a fast stretching can produce a high reflex activity. Related scientific considerations were found regarding reflective effects during the SSC, although there were not starting from an isometric muscle tension.

An activation frequency could be taken into account.

Within our setting none of the assumed possible mechanisms can be proved, since no electromyographical activity was detected. Further research has to implement appropriate recordings.

### Possible Applications in Exercise

It is supposed here, that the “boosting” effect might be useful in exercise. If this effect could be supported by further investigations, a training using the xpAF could probably be effective because of the following reasons. Due to the higher resistance, which was achieved during xpAF with higher pre-activation, we assume that higher training intensities could be reached. In this case, we might expect higher training effects for athletes.

It already is known that eccentric training can be highly effective concerning hypertrophy, power and rehabilitation after injuries (e.g., Magnussen et al., 2009; Vogt and Hoppeler, 2014; Coratella and Schena, 2016). Several authors propose that during eccentric action a neural inhibition occurs, since the EMG amplitude decreases comparing to concentric (e.g., Westing et al., 1990; Aagard et al., 2000; Linnamo, 2002; Linnamo et al., 2003). Duchateau and Baudry (2014) suggest that the specific neural adjustment associated with eccentric action may explain, why eccentric training seems to be so promising.

Since mostly slow eccentric training is investigated, the authors see an additional potential in high-velocity eccentric training with a preload of at least 30% of the MVIC. This showed an even higher force outcome and, therefore, higher intensities could be achieved. Furthermore, several investigators concluded that fast eccentric training is most effective for muscle hypertrophy and strength comparing different velocities or in comparison with concentric exercise (e.g., Farthing and Chilibeck, 2003; Shepstone et al., 2005).

Since the measurement with the here used pneumatic system enables the participant to adapt to the external force impact, the authors assume an advantage comparing to other systems, esp. isokinetic systems – also in training. This motion is closer to real life movements and thus, possibly, the adaptation to external force impacts is additionally trained. In sports, the organism always has to adapt to external influences. According to Vogt and Hoppeler (2014), muscles function also as shock absorbers during lengthening movements, to slow down landing tasks or to precisely handle high external loading in sports (e.g., alpine skiing). The AF covers the mechanisms of adaptation to external impacts. Since the strength benefits from eccentric training seems to be highly specific to the mode of muscle action and the velocity of movement (Roig et al., 2009), exercise using the xpAF could train this specific capability of adaptation during quick movements with high loads. This may additionally have a preventive effect with respect to sport injuries.

## Conclusion

The findings of the present study could lead to new insights regarding eccentric muscle action and exercise. The hypothesis that training using the xpAF with an optimized muscular pre-activation has effects on strength, power and/or hypertrophy must be investigated. If further investigations confirm this hypothesis, the authors anticipate the main application in power performance of top athletes. Furthermore, this could bring strength and power training closer to natural muscle actions, which might have an injury-preventing effect.

## Ethics Statement

The study was carried out in accordance with the recommendations of the ethical committee, University of Potsdam, Germany. The protocol was proofed by the local ethical committee of the University of Potsdam, Germany. All subjects gave written informed consent in accordance with the Declaration of Helsinki.

## Author Contributions

Both authors contributed to the conception and design of the study, participated in the process of interpretation and discussion of the data, critically revised the manuscript, and read and approved the submitted version of the manuscript. LS performed the acquisition and analysis of the data, and drafted the manuscript.

## Conflict of Interest Statement

The authors declare that the research was conducted in the absence of any commercial or financial relationships that could be construed as a potential conflict of interest.

## Abbreviations

AF: Adaptive Force
EMG: electromyography
MVC: maximal voluntary contraction
MVIC: maximal voluntary isometric contraction
PAP: post-activation potentiation
SD: standard deviation
SSC: stretch shortening cycle
xpAF: explosive Adaptive Force
xpAFecc_max_: maximal explosive eccentric Adaptive Force.
